# Radial extracorporeal shock wave therapy is efficient and safe in the treatment of fracture nonunions of superficial bones: a retrospective case series

**DOI:** 10.1186/s13018-017-0667-z

**Published:** 2017-11-06

**Authors:** Paulo Kertzman, Nikolaus B. M. Császár, John P. Furia, Christoph Schmitz

**Affiliations:** 1 Departamento de Ortopedia, Santa Casa de São Paulo, São Paulo, SP Brazil; 2 Extracorporeal Shock Wave Research Unit, Chair of Neuroanatomy, Institute of Anatomy, Faculty of Medicine, LMU Munich, Pettenkoferstr 11, D-80336 Munich, Germany; 3 SUN Orthopaedics and Sports Medicine, Division of Evangelical Community Hospital, 900 Buffalo Road, Lewisburg, PA 17837 USA

**Keywords:** Bone, Focused extracorporeal shock wave therapy, Fracture, Nonunion, Radial extracorporeal shock wave therapy

## Abstract

**Background:**

A substantial body of evidence supports the use of focused extracorporeal shock wave therapy (fESWT) in the non-invasive treatment of fracture nonunions. On the other hand, virtually no studies exist on the use of radial extracorporeal shock wave therapy (rESWT) for this indication.

**Methods:**

We retrospectively analyzed 22 patients treated with rESWT for fracture nonunions of superficial bones that failed to heal despite initial surgical fixation in most cases. Radial extracorporeal shock wave therapy was applied without anesthesia in three rESWT sessions on average, with one rESWT session per week and 3000 radial extracorporeal shock waves at an energy flux density of 0.18 mJ/mm^2^ per session. Treatment success was monitored with radiographs and clinical examinations.

**Results:**

Six months after rESWT radiographic union was confirmed in 16 out of 22 patients (73%), which is similar to the success rate achieved in comparable studies using fESWT. There were no side effects. The tibia was the most common treatment site (10/22) and 70% of tibia nonunions healed within 6 months after rESWT. Overall, successfully treated patients showed a mean time interval of 8.8 ± 0.8 (mean ± standard error of the mean) months between initial fracture and commencement of rESWT whereas in unsuccessfully treated patients the mean interval was 26.0 ± 10.1 months (*p* < 0.05). In unsuccessful tibia cases, the mean interval was 43.3 ± 13.9 months.

**Conclusions:**

Radial extracorporeal shock wave therapy appears to be an effective and safe alternative in the management of fracture nonunions of superficial bones if diagnosed early and no fESWT device is available. The promising preliminary results of the present case series should encourage the implementation of randomized controlled trials for the early use of rESWT in fracture nonunions.

**Electronic supplementary material:**

The online version of this article (10.1186/s13018-017-0667-z) contains supplementary material, which is available to authorized users.

## Background

Nonunion refers to the failure of bone fractures to achieve cortical continuity on radiographic studies. The prevalence of nonunion of all fracture types ranges from 2.5 to 46%, and the complication places substantial economic burdens on health systems [1–3]. Surgical fracture stabilization using bone grafts and internal/external fixation has remained the gold standard for treating fracture nonunions. However, these procedures often lead to serious complications including deep infections, persistent wound drainage, hematoma formation, sensory loss, persisting pain, and nonunions [2, 4–6].

Hence, there remains a need for efficient therapies that will bring better results more quickly and without major complications. Over the past decades, extracorporeal shock wave therapy (ESWT) has emerged as an efficient, non-invasive and cost-effective alternative to surgery in the treatment of fracture nonunions. There are two forms of ESWT available, focused (fESWT) and radial (rESWT) (see [7–9] for detailed reviews). In very brief, fESWT makes use of single acoustic pulses that are generated either with a spark-gap (electrohydraulic principle), a technology similar to a loudspeaker (electromagnetic principle), or piezocrystals (piezoelectric principle). By means of reflectors of certain shape, the acoustic pulses are converted into a focused acoustic pressure wave/shock wave with a point of highest pressure at the desired target within pathological tissue. In case of rESWT, a projectile is fired within a guiding tube that strikes a metal applicator placed on the skin. The projectile generates stress waves in the applicator that transmit pressure waves into tissue. Both focused and radial extracorporeal shock waves are single acoustic impulses with an initial high positive peak pressure between 10 and 100 MPa reached in less than 1 μs. The positive pressure amplitude is followed by a low tensile amplitude of a few microseconds duration that can generate cavitation. They are further characterized by a short life cycle of approximately 10–20 μs and a broad frequency spectrum (see [7–9] for more details).

A total of 40 studies [10–49] have shown an overall success rate of approximately 76% after 6 months when treating fracture nonunions with fESWT, without major complications (Table 1; a comprehensive overview on details of these studies is provided in Additional file 1). Among these 40 studies were 1 randomized controlled trial (RCT) with a success rate of 71% after 6 months [10], 2 cohort studies comparing fESWT with surgery, with success rates of 91 [11] and 79% [12] after 6 months, and 37 case series without control group [13–49]. The RCT [10] and the cohort studies [11, 12] were recently reviewed in detail elsewhere [50].

**Table 1:** Overview on all studies investigating the effects of focused extracorporeal shock wave therapy (fESWT) for fracture nonunions listed in PubMed (as of March 01, 2017)

R	Study	*T*	*n*	*D*	Interval [months]	SR_M6_ [%]	SR_T_ [%]	*N*_S_	Nf_ESWs/S_	EFD [mJ/mm^2^]	EFD_T_ [mJ/mm^2^]	Relative EFD_T_	KV
[10]	Cacchio et al. (2009)	RCT	84	EM	11.1	71	94	4	4000	0.55	8800	12.1	
[11]	Furia et al. (2010)	CH	23	EH	10.4	91	91	1	3000	0.35	1050	1.4	26
[12]	Notarnicola et al. (2010)	CH	58	EM	14.8	79	79	3	4000	0.09	1080	1.5	
[13]	Valchanou and Michailov (1991)	CS	79	EH	20.2		85	1	2500				
[14]	Schleberger and Senge (1992)	CS	4	EH	≥ 5	75	75	1	2000				18
[15]	Heinrichs et al. (1993)	CS	53	EM			67	1	5750				
[16]	Diesch & Haupt (1997)	CS	172	EH, EM		66	66	1	2500	0.33	813	1.1	
[17]	Haupt (1997)	CS	100	EH			65						
[18]	Haupt (1997)	CS	87	EH			67	1	2000				21
[19]	Vogel et al. (1997)	CS	52				52						
[20]	Vogel et al. (1997)	CS	48	EM	12		60	1	3000	0.6	1800	2.5	
[21]	Beutler et al. (1999)	CS	25	EH	9	41	41	2	2000				18
[22]	Rompe et al. (2001)	CS	43	EM	11.4		72	1	3000	0.6	1800	2.5	
[23]	Schaden et al. (2001)	CS	115	EH	6		76	1	6500	0.33	2113	2.9	24
[24]	Wang et al. (2001)	CS	72	EH		61	80	1	3500	0.55	1908	2.6	
[25]	Küfer et al. (2002)	CS	4	EM	≥ 6		75	3	2500	0.12	900	1.2	
[26]	Schatz et al. (2002)	CS	31	EM	10.5		68	1	6000	1.5	9000	12.3	
[27]	Biedermann et al. (2003)	CS	73	EH	6		56	1	2900	0.7	2030	2.8	
[28]	Chooi and Penafort (2004)	CS	5	EH	26.6	40	40	1	4000				25
[29]	Schaden et al. (2004)	CS	613	EH	16.1	76	76	1	3000	0.38	1140	1.6	
[30]	Bara and Synder (2007)	CS	81	EH	8	83	83	1	2250				20
[31]	Taki et al. (2007)	CS	5	EH	12	100	100	1	3000	0.35	1050	1.4	25
[32]	Endres et al. (2008)	CS	1	EM	9	100	100	4		0.4			
[33]	Cacchio et al. (2009)	CS	34	EM	6	77	77	4	4000	0.4	6400	8.8	
[34]	Moretti et al. (2009)	CS	204	EM		85	85	1	4000	0.66	2640	3.6	
[35]	Wang et al. (2009)	CS	42	EH	15	79	79	1	6000	0.62	3720	5.1	28
[36]	Xu et al. (2009)	CS	69	EM	12.5	65	76	1	6500	0.59	3835	5.3	26
[37]	Elster et al. (2010)	CS	192	EH	16.8	72	72	1	7000	0.39	3100	4.2	27
[38]	Alvarez et al. (2011)	CS	32	EH	7	73	95	1	2000	0.37	730	1	
[39]	Stojadinovic et al. (2011)	CS	349	EH		81	81	1	7000	0.5	3500	4.8	27
[40]	Vulpiani et al. (2012)	CS	143	EM	14.1		56	6	2750	0.55	8301	11.2	
[41]	Czarnowska-Cubała et al. (2013)	CS	31	EH	22.6	39	39	1	3000				20.5
[42]	Alkhawashki (2015)	CS	44	EH	11.9		76	1	3000				26
[43]	Kuo et al. (2015)	CS	22	EH	10.5		64	1	6000	0.58	3480	4.8	28
[44]	Haffner et al. (2016)	CS	52	EH	15.6	89		1	4000	0.4	1600	2.2	
[45]	Ikeda et al. (1999)	CS	6	^a^	14		67						
[46]	Ikeda (2009)	CS	8	^a^			63						

*R* reference number, *T* type of study, *RCT* randomized controlled trial, *CH* cohort study, *CS* case series, *n* number of patients treated with fESWT, *D* type of fESWT device, *EH* electrohydraulic fESWT device, *EM* electromagnetic fESWT device, *interval* interval between initial fracture and first nonunion treatment (fESWT or other), *SR*_*M6*_ success rate after 6 months, *SR*_*T*_ total success rate, *N*_*S*_ number of fESWT sessions, *N*_*fESWs/S*_ number of focused extracorporeal shock waves per session, *EFD* energy flux density of the applied fESWs, *EFD*_*T*_ total energy flux density, *relative EFD*_*T*_ multiple of EFD_T_ compared to the EFD_T_ applied in [38], *KV* kilovolt. Note that for *N*_*S*_, N_fESWs/S_, EFD, EFD_T_, and KV average values are provided in case more than one fESWT protocol was used in the corresponding study (details are provided in Additional file 1). In case no data are shown, they were either not provided in the corresponding study or could not be calculated (details are provided in Additional file 1). Note that the following studies are not listed in the table: [47] (same data as in [19]), [48] (same data as in [22]) and [49] (dataset included in [30])

^a^Extracorporeal shock waves generated by means of explosions

In contrast, there are only few reports of using rESWT for treating fracture nonunions. We are aware of only one case report study demonstrating the success of rESWT in the consolidation of fracture nonunions involving the base of the second metatarsal in two professional dancers [51].

Acknowledging recent findings (i) from an animal study in vivo demonstrating that radial extracorporeal shock waves (rESWs) can induce new bone formation [52] as well as (ii) from an in vitro study showing that rESWs can induce proliferation of human osteoblast like cells (MG63) [53], the purpose of the present study was to test the following hypothesis: radial extracorporeal shock waves can stimulate bone healing in fracture nonunions of superficial bones.

## Methods

The present study represents a retrospective case series in 22 patients investigating the effects of rESWT on fracture nonunions between 2007 and 2012. The study was approved by the ethics committee of the Medical School of Santa Casa de São Paulo (São Paulo, Brazil) (Reference numbers 12558513.1.0000.5479 CAAE and 232.292 from 27 March 2013). Informed consent was achieved from each patient to participate in this study.

In line with several reports on fESWT for facture nonunions in the literature [11, 37, 39, 43], a nonunion was defined as a fracture that has failed to show continuity of three of four cortices after surgical or nonsurgical treatment for six or more months from the time of the fracture-related injury, or has failed to demonstrate any radiographic change (improvement) for three consecutive months, and is associated with clinical findings consistent with a fracture nonunion (an inability to bear weight on the affected extremity, pain on palpation, or motion at the fracture site for 3 to 6 months or more following the incident traumatic event or the last surgical procedure. Patients included 9 females and 13 males, with a mean age of 35.3 ± 3.5 years (mean ± standard error of the mean) (range 14 to 69) (Table 2). Twenty of these patients had undergone initial surgical treatment of the fracture and presented to the first author of the present study at different time points postoperatively (months to years) due to persisting pain and functional limitations. Fracture nonunion sites in these patients included the clavicle (i.e., collarbone; one patient), ulna (one patient), carpal scaphoid (one patient), tibia (10 patients, one of whom with an infected/atrophic tibia), medial malleolus (two patients), lateral malleolus (one patient), navicular bone of the foot (one patient), second metatarsal bone (one patient), and fifth metatarsal bone (two patients). Surgical procedures included internal plates, nails, and intramedullary/internal screw fixations. The remaining two patients, one with an inferior iliac crest fracture and one with a fibula fracture, presented to the first author of the present study, respectively, 7 and 8 months after the trauma without receiving initial surgery.

**Table 2:** Clinical data and treatment outcome of patients with fracture nonunions enrolled in the present study

Case #	Sex	Age (years)	Nonunion site	Fixation	*I*	*S*	*O*
1	M	38	Ulna	Internal plate	7	4	**+**
2	M	30	Carpal scaphoid	Screw fixation	5	3	**+**
3	F	14	Tibia	Intramedullary nail (locked)	12	4	**+**
4	F	21	Tibia	Intramedullary nail (locked)	9	5	**+**
5	F	28	Tibia	Intramedullary nail (locked)	7	3	**+**
6	F	43	Tibia and fibula	Intramedullary nail (tibia, locked)	5	4	**+**
7	M	46	Tibia	Intramedullary nail (locked)	10	2	**+**
8	M	48	Tibia	Intramedullary nail (locked)	9	3	**+**
9	M	59	Tibia	Intramedullary nail (locked)	10	4	**+**
10	F	58	Medial malleolus	Screw fixation	14	3	**+**
11	M	69	Medial malleolus	Screw fixation	6	3	**+**
12	F	15	Second metarsal	Screw fixation	6	4	**+**
13	M	19	Fifth metatarsal	Screw fixation	17	3	**+**
14	M	20	Fifth metatarsal	Screw fixation	9	3	**+**
15	M	15	Iliac crest	n.a.	7	3	**+**
16	M	21	Fibula	n.a.	8	4	**+**
17	F	53	Clavicle	Internal plate	4	2	**–**
18	F	19	Tibia	Intramedullary nail (locked)	18	5	**–**
19	M	33	Tibia (infected)	Intramedullary nail (locked), internal plate	66	6	**–**
20	M	37	Tibia	Intramedullary nail (locked)	46	5	**–**
21	F	49	Lateral malleolus	Srew fixation	6	3	**–**
22	M	41	Navicular bone (foot)	Screw fixation	16	3	**–**

*I* interval between fracture and first session of radial extracorporeal shock wave therapy (rESWT), *S* number of rESWT sessions, *O* outcome, + positive clinical outcome, − negative clinical outcome

All patients received their first rESWT treatment between May 2007 and September 2012. The standard procedure for rESWT in the present study consisted of three consecutive outpatient clinic rESWT sessions in 1-week intervals, although the number of rESWT sessions was adjusted to each patients’ individual clinical situation (Table 2). Radial ESWT was performed without anesthesia using a Swiss DolorClast device (Electro Medical Systems, Nyon, Switzerland) and consisted of 3000 radial extracorporeal shock waves per session at the device’s highest energy setting (i.e., 4 bar air pressure equivalent to an energy flux density, EFD, of 0.18 mJ/mm^2^). The “radial” handpiece of the device with its 15-mm applicator was used in all cases. In each case, the point of application was determined prior to rESWT by means of radiographs and palpation. After rESWT, patients with metatarsal fractures were advised to wear an open toe cast shoe for 3 weeks whereas patients with tibia fractures were advised to refrain from weight bearing and any sports activities for 4 weeks. During the following 6 months patients were monitored by means of radiographs and clinical examinations.

A positive therapy outcome was defined by radiographic consolidation and absence of both pain and functional limitations during normal weight loading 6 months after commencement of rESWT. Adverse events during and after rESWT were documented.

Mean and standard error of the mean (SEM) of patient’s age, number of rESWT sessions, and interval between the initial fracture and the first rESWT session were separately calculated for patients with positive clinical outcome (Group rESWT+) and patients with negative clinical outcome (group rESWT-). The Shapiro-Wilk normality test was used to determine whether the distribution of patients’ age, the interval between the initial fracture and the first rESWT session, and the number of rESWT sessions of the patients in Groups rESWT+ and rESWT- were consistent with a Gaussian distribution. Differences between groups were tested with (i) Fisher’s exact test for the relative numbers of female and male patients, (ii) Student’s *t* test for the mean age of the patients and the mean interval between the initial fracture and the first rESWT session, and (iii) non-parametric Mann Whitney test for the mean number of rESWT sessions. In all analyses, an effect was considered statistically significant if its associated *p* value was smaller than 0.05. All calculations were performed using GraphPad Prism (version 5.00 for Windows, GraphPad Software, San Diego, CA, USA).

## Results

Six months after receiving rESWT, 16 out of 22 patients (16/22 = 73%) had a positive outcome defined by radiographic bone consolidation and the absence of both pain and functional limitations during normal weight loading. Treatments were well tolerated by all patients, with bearable pain during treatment devoid of anesthesia being the main complication reported. Neither skin injuries nor hematomas nor ecchymosis were observed during or after any of the rESWT sessions.

**Fig. 1:**
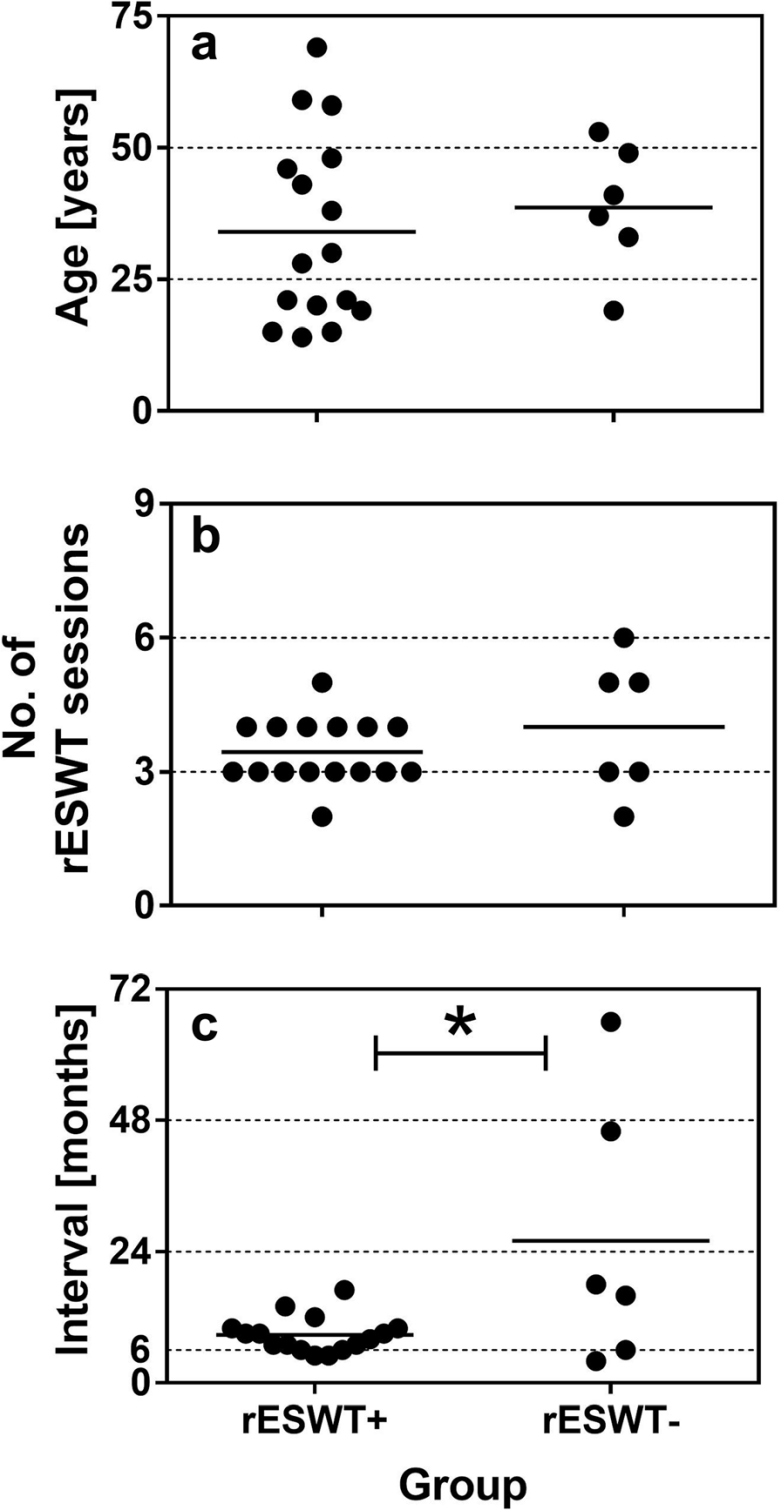
Individual data (dots) and mean values (horizontal lines) of the patients’ age (**a**), number of rESWT sessions (**b**), and interval between initial fracture and the first rESWT session (**c**) of patients with positive clinical outcome (Group rESWT+) and patients with negative clinical outcome (group rESWT−). *p < 0.05

There were no statistically significant age or gender differences observed among patients with positive outcome (average age: 34.0 ± 4.5 years; mean ± SEM; *n* = 16; 6 females, 10 males) or negative outcome (average age: 38.7 ± 5.0 years; *n* = 6; three females and three males) (age: *p* = 0.565; gender: *p* = 0.655) (Fig. 1a). Nor was there a statistically significant difference in the mean number of rESWT sessions applied between the groups with positive (3.4 ± 0.2 sessions) and negative outcome (4.0 ± 0.6 sessions) (*p* = 0.253) (Fig. 1b). On the other hand, there was a statistically significant difference between Groups rESWT+ and rESWT− with respect to the time interval between initial trauma and first application of rESWT: in the group with positive clinical outcome this time interval was 8.8 ± 0.8 months whereas in the group with negative clinical outcome on average 26.0 ± 10.1 months had passed between bone fracture and rESWT commencement (*p* = 0.010) (Fig. 1c).

**Fig. 2:**
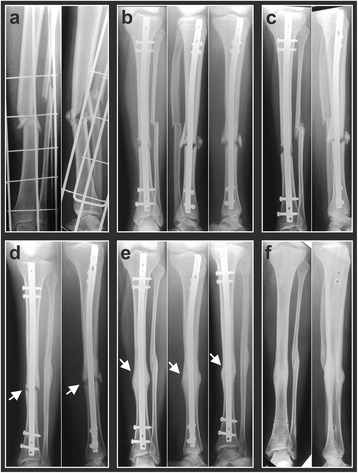
Radiographs of a 14-year-old-girl who suffered in a traffic accident a combined fracture of the left tibia and fibula, showing the situation immediately after the accident (**a**) as well as 2 weeks (**b**), 3 months (**c**), and 12 months (**d**) after the accident. Fracture consolidation was still not achieved (arrows in **d**), and the patient experienced severe pain during walking. At that time, a series of four rESWT sessions was started. Six months later (**e**), the patient was pain-free during walking, and radiographic consolidation was achieved (arrows in **e**). Another 12 months later, the intramedullary nail was removed (**f**)

In the present study, the tibia was the most frequent fracture nonunion site, and in 7 out of 10 tibia cases (7/10 = 70%) rESWT yielded positive results. An impressive case is shown in Fig. 2. This was a 14-year-old girl who suffered in a traffic accident a combined fracture of the left tibia and fibula (Fig. 2a). Initial surgery was performed using locked intramedullary nailing of the tibia (Fig. 2b). Twelve months after the initial fracture consolidation was still not achieved, and the patient experienced severe pain during walking (Fig. 2c, d). At this time point, a series of four rESWT sessions was started. Six months later, the patient was pain-free during walking, and radiographic consolidation was achieved (Fig. 2e). Another 12 months later the intramedullary nail was removed (Fig. 2f).

Regarding those tibia patients unresponsive to rESWT, it is noteworthy that their time interval between fracture and rESWT commencement was on average approximately five times higher compared to the group with positive clinical outcome (i.e., 43.3 ± 13.9 months vs. 8.9 ± 0.9 months, respectively). Furthermore, one of the unsuccessfully treated patients showed a tibia atrophy together with a medical history comprising eight failed previous surgeries during an interval of 66 months, the longest interval among all patients in the present study.

The remaining nine cases with positive clinical outcome involved the ulna (one patient), carpal scaphoid (one patient), inferior iliac crest (one patient), fibula (one patient), medial malleolus (two patients), second metatarsal bone (one patient), and fifth metatarsal bone (two patients). In contrast, in one of each case involving the clavicle, lateral malleolus and navicular bone of the foot rESWT failed to achieve bony union for unknown reasons.

## Discussion

The present retrospective study, to our knowledge, represents the first pilot case series involving more than two patients with fracture nonunions treated with rESWT. Sixteen out of 22 patients (73%) were successfully treated with rESWT, as confirmed by radiographic consolidation at the 6-month follow-up. There were no complications. Anesthesia during treatment was not necessary. Nonunions of the tibia were the most common and 70% of the tibia nonunions healed within 6 months after rESWT.

Both focused and radial extracorporeal shock waves are single acoustic impulses with an initial high positive peak pressure between 10 and 100 MPa reached in less than 1 μs (reviewed in [9]). The positive pressure amplitude is followed by a low tensile amplitude of a few microseconds duration that can generate cavitation [54–56]. They are further characterized by a short life cycle of approximately 10–20 μs and a broad frequency spectrum [57]. Focused ESWs differ from rESWs in the penetration depth into the tissue, some physical characteristics, and the technique for generating them [7, 9, 57].

We noted an association between prolonged time interval between fracture and rESWT procedure and failure of treatment. This discrepancy among groups with positive and negative clinical outcome was most pronounced in tibia cases, where unsuccessfully treated patients displayed on average approximately five times longer intervals than successfully treated patients. In this respect, it is important to point out that the interval (together with the specific type of bone affected) has been identified as the most predictive prognostic indicator for fracture nonunion healing at 6 months following fESWT [39]; i.e., with intervals longer than 11 months significantly reducing the likelihood of a positive clinical outcome (see also [44]). In the present study, the mean interval among the few rESWT resistant patients and particularly among therapy resistant tibia patients exceeded this 11-month threshold, respectively, two- and fourfold. The interval is therefore of relevance when treating fracture nonunions with ESWT, both in clinical routine and in future RCTs on this topic.

Some reports in the literature [44, 58–60] referred to the following definition of fracture nonunion provided by the United States Food and Drug Administration (FDA) [61]: “A non-union study would include patients whose fractures have not healed for a minimum of nine months and who have not undergone surgical intervention during the previous three months.” It is important to note that only two out of the 37 studies (5.4%) on fESWT for fracture nonunions listed in Table 1 [22, 44] adhered to this definition. In contrast, in 22 of these studies (59.5%) a minimum time interval of 6 months was applied to define fracture nonunions (as was done in the present study and in the only prior report involving the use of rESWT for treating fracture nonunions [51] discussed below). Furthermore, in 9 out of the 37 studies (24.3%) listed in Table 1, no definition of fracture nonunions was provided, and in four of these studies different definitions of fracture nonunions were applied (details are outlined in Additional file 1). Several reasons for this discrepancy are conceivable. Specifically, the definition by FDA was provided without any reference to the academic literature and was provided in a draft of a Guidance Document that was released for comment in 1998 but has never become an official FDA Guidance Document. Accordingly, the definition provided above may have never become official opinion by FDA.

To our knowledge, there exists only one prior report involving the use of rESWT for treating fracture nonunions [51]. In this small case series of two professional dancers, rESWT was successfully used to achieve fracture union of the base of the second metatarsal bone. One fracture nonunion healed 3 months post treatment and the other healed 6 months post treatment. In the present study, the same treatment protocol as used by Silk et al. [51] was applied (i.e., three rESWT sessions at 1-week intervals; 3000 radial extracorporeal shock waves per session), although with a slightly lower energy flux density (i.e., 0.18 vs. 0.20 mJ/mm^2^), and positive clinical outcome was achieved in the majority of cases. Therefore, rESWT appears to be an effective and safe treatment option for fracture nonunions of superficial bones in case a fESWT device is not available.

With exception of one study [51], all clinical studies on the effects of ESWT in the treatment of fracture nonunions have so far been conducted using fESWT (Table 1) [10–49]. This evidence listed in PubMed as of 01 March 2017 includes one prospective RCT (level of evidence, 1) comparing two different fESWT protocols with surgery [10], 2 cohort studies also comparing fESWT with surgery [11, 12], 34 case series without control group [13–46], and 3 double publications of data [47–49]. Collectively, these 40 studies have shown a mean success rate of approximately 76% after 6 months. However, this mean success rate must be interpreted cautiously because of substantial heterogeneity of these studies with respect to the treatment site and the fESWT protocol used (note that in some studies [17, 18, 25, 34], the treatment site was not specified). The success rate reported in the RCT comparing fESWT with surgery [10] cannot directly be compared to the results of the present study because in the RCT, 53% of the treatment sites (67/126) were the tibia (48% in the present study) but 27% (34/126) were the femur, 12% (15/126) were the ulna, and 8% (10/126) were the radius (femur and radius were not addressed in the present study). The same applies to the success rates reported in the cohort studies comparing fESWT with surgery because in these studies, only patients suffering from fracture nonunions of, respectively, the proximal fifth metatarsal [11] (only two patients in the present study) or the carpal scaphoid [12] (only one patient in the present study) were included. On the other hand, Elster et al. [37] reported for 192 fracture nonunions of the tibia a success rate of 72% at 6 months after fESWT, which is close to the success rate reported in the present study. Very recently, Haffner et al. [44] reported for 52 fracture nonuions of the tibia a success rate of 89% at 6 months after fESWT, which is higher than the success rate reported in the present study.

**Fig. 3:**
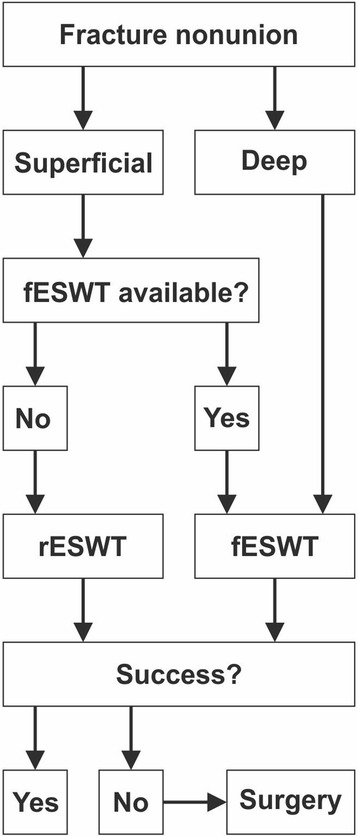
Proposed decision tree for the treatment of fracture nonunions with extracorporeal shock waves based on the evidence published so far. fESWT focused extracorporeal shock wave therapy, rESWT radial extracorporeal shock wave therapy

Based on the evidence outlined above, it appears reasonable to propose a “therapy of first choice” recommendation for the treatment of fracture nonunions using ESWT (Fig. 3). Beforehand, it must be stressed that ESWT for nonunions, regardless of whether fESWT or rESWT, must be considered a medical treatment to be performed by medical doctors with exact knowledge about orthopedic diagnostic of nonunion and capacity to decide the best treatment option and possible complications. This is because pain relief must not be used as sole clinical endpoint in these cases; mainly but not exclusively due to the analgesic effects of ESWT, usually causing patients to mobilize the affected limb prior to achieving bony consolidation. The recommendation outlined in Fig. 3 considers both superficial and deep as well as early and late fracture nonunions. In either of these scenarios, as soon as possible after correct fracture nonunion diagnosis, early fESWT if available should be chosen over rESWT or surgery. If fESWT is not available and the bone with the fracture nonunion is superficial, early rESWT instead of surgical procedures should be opted for. If fESWT or rESWT is not successful in treating fracture nonunions of superficial bones then surgical procedures should be performed.

This recommendation seems to be justified not only from the preliminary evidence presented in the present study but also from the increasing evidence stemming from concerted basic research efforts into the mechanisms of rESWT over the last years: (i) the application of rESWT in deep indications such as nonunions of the femur or avascular hip necrosis is contraindicated simply because rESWs cannot reach the intended tissues [55]. On the other hand, radial extracorporeal shock waves can reach superficial bones (including those listed in Table 2), and the results of the present study suggest that rESWT is a safe and effective treatment for fracture nonunions of superficial bones. (ii) For both rESWs and fESWs, new bone formation was demonstrated in animal models in vivo [52, 62]. (iii) For both rESWs and fESWs, induction of proliferation of human osteoblast like cells or primary human osteoblasts was shown in vitro [53, 63].

The present study is an audit of retrospectively collected data and has therefore inherent limitations. First, there was no randomization and no surgery arm to this study; however, the same was the case in 92% of all studies on fESWT for fracture nonunions (34 out of 37 studies without double publication of data). Second, the small number of patients could potentially confound the clinical results. Third, treatment success was determined at 6 months after rESWT, i.e., no total success rate of rESWT (regardless of the follow-up interval) for fracture nonunions could be determined. Fourth, no test of other imaging modalities of fracture nonunions such as computer tomography or scintigraphy was performed; however, the symptoms and imaging findings used to define positive clinical outcome in the present study are generally accepted and considered appropriate for this condition.

## Conclusions

The results of the present retrospective study suggest that rESWT is a safe and effective treatment for fracture nonunions of superficial bones. For this reason, medical doctors should consider rESWT prior to surgical intervention in the management of fracture nonunions of superficial bones in case fESWT is not available. In any case, ESWT should be performed as soon as possible after correct diagnosis of fracture nonunion. The promising results of the present retrospective study should encourage the implementation of RCTs using early rESWT in the treatment of fracture nonunions of superficial bones.

## Additional file

Additional file 1: Details of all studies on focused extracorporeal shock wave therapy (fESWT) for fracture nonunions listed in PubMed (as of March 01, 2017). (DOCX 76 kb)

## Abbreviations

CH: Cohort study
CS: Case series
D: Type of fESWT device
EFD: Energy flux density
EFD_T_: Total energy flux density
EH: Electrohydraulic fESWT device
EM: Electromagnetic fESWT device
ESWT: Extracorporeal shock wave therapy
FDA: United States Food and Drug Administration
fESWs: Focused extracorporeal shock waves
fESWT: Focused extracorporeal shock wave therapy
I: Interval between fracture and first session of radial extracorporeal shock wave therapy
KV: Kilovolt
mJ/mm^2^: millijoule per squared millimeter
MPa: Megapascal
n: Number of patients treated with fESWT
n.a.: Not applicable
N_fESWs/S_: Number of focused extracorporeal shock waves per session
N_S_: Number of fESWT sessions
O: Outcome
R: Reference number
RCT: Randomized controlled trial
rESWs: Radial extracorporeal shock waves
rESWT-: Patients with negative clinical outcome after rESWT
rESWT: Radial extracorporeal shock wave therapy
rESWT+: Patients with positive clinical outcome after rESWT
S: Number of sessions
SEM: Standard error of the mean
SR_M6_: Success rate after 6 months
SR_T_: Total success rate
T: Type of study
μs: Microsecond
