# Supplementary material for: Radial extracorporeal shock wave therapy is efficient and safe in the treatment of fracture nonunions of superficial bones: a retrospective case series
Source: J Orthop Surg Res. 2017 Nov 6;12:164. doi: 10.1186/s13018-017-0667-z (PMC5674749; doi:10.1186/s13018-017-0667-z)
Supplement: Additional file 1: — Details of all studies on focused extracorporeal shock wave therapy (fESWT) for fracture nonunions listed in PubMed (as of March 01, 2017). (DOCX 76 kb) [file 13018_2017_667_MOESM1_ESM.docx]

**Radial extracorporeal shock wave therapy for the treatment of fracture nonunions: a retrospective case series**

by Paulo Kertzman, Nikolaus B.M. Császár, John P. Furia and Christoph Schmitz

**Additional file 1: details of all studies on focused extracorporeal shock wave therapy (fESWT) listed in PubMed (as of March 01, 2017)**

All data are provided as mean ± standard deviation

fESWT, focused extracorporeal shock wave therapy

fESWs, focused extracorporeal shock waves

Interval, interval between initial fracture and first nonunion treatment (fESWT or other)

Follow-up, interval between fESWT and follow-up examination(s)

**1) Randomized controlled trials**

| **Reference no.** | [1] |
| --- | --- |
| **Reference** | Cacchio et al. (2009a) |
| **Type** | Randomized controlled trial |
| **Patients** | 126 total  ESWT group I: 42 (10♀, 32♂); mean age 42.8 ± 6.3 years  ESWT group II: 42 (11♀, 31♂); mean age 43.1 ± 5.4 years  surgery group: 42 (12♀, 30♂); mean age 42.5 ± 6.2 years |
| **Definition of nonunion** | “A fracture that did not show any progress toward healing on radiographs made at  one-month intervals for at least six months following treatment.” |
| **Treatment sites** | 126 including tibia (67), femur (34), ulna (15) and radius (10) |
| **fESWT protocol / comparator** | ESWT groups: four sessions @ one-week intervals; 4,000 fESWs @ 0.40 mJ/mm^2^ in group I, and 0.70 mJ/mm^2^ in group II;  Comparator group: locked intramedullary nail alone (23) or combined with autogenous bone graft (12), external fixation (7) |
| **fESWT device** | Group I : Epos Ultra (electromagnetic) (Dornier MedTech, Wessling, Germany)  Group II: Modulit SLK (electromagnetic) (Storz Medical, Tägerwillen, Switzerland) |
| **Anesthesia** | ESWT groups I and II: regional  Surgery group: general |
| **Interval** | ESWT group I: 11.5 ± 4.9 months  ESWT group II: 10.8 ± 5.4 months  Surgery group: 10.2 ± 6.1 months |
| **Follow-up** | 3, 6, 12 and 24 months |
| **Outcome** | Equal to comparator  ESWT group I: union in 22/40 (55%) of patients after 3 months, in 26/37 (70%) after 6 months, in 31/37 (84%) after 12 months and in 34/36 (94%) after 24 months.  ESWT group II: union in 21/39 (54%) of patients after 3 months, in 22/38 (71%) after 6 months (=I.E.P.), in 31/38 (82%) after 12 months and in 35/38 (92%) after 24 months.  Surgery group: union in 21/40 (52%) of patients after 3 months, in 28/38 (74%) after 6 months, in 33/38 (87%) after 12 months and in 35/37 (95%) after 24 months. |

**2) Cohort studies (comparing fESWT with surgery)**

| **Reference no.** | [2] |
| --- | --- |
| **Reference** | Furia et al. (2010) |
| **Type** | Case-control study |
| **Patients** | 43 total  ESWT group: 23 (10♀, 13♂), mean age 42.7 ± 18.0 (17-78) years  surgery group: 20 (12♀, 8♂), mean age 40.8 ± 18.6 (19-78) years |
| **Definition of nonunion** | “A fracture that had failed to demonstrate cortical continuity radiographically for six months despite operative or nonoperative intervention or that had shown no radiographic evidence of healing for three months and was associated with pain  and/or tenderness on palpation.” |
| **Treatment sites** | 43 proximal fifth metatarsal metaphyseal-diaphyseal fractures |
| **fESWT protocol / comparator** | ESWT group: single session; 2,000-4,000 fESWs @ 26 kV (0.35 mJ/mm^2^)  Comparator group: intramedullary screw fixation |
| **fESWT device** | Ossatron (electrohydraulic) (HMT, Lengwil, Switzerland) and Orthowave 280 (electrohydraulic) (MTS, Konstanz, Germany) |
| **Anesthesia** | ESWT group: general (15), regional (6) or local (2)  Surgery group: not specified |
| **Interval** | ESWT group 10.4 ± 7.0 months (range, 6-39)  Surgery group 6.2 ± 2.3 months (range, 4-14) |
| **Follow-up** | 6 months |
| **Outcome** | Equal to comparator  ESWT group: union in 21/23 (91.3%) of patients  Surgery group: union in 18/20 (90%) of patients |

| **Reference no.** | [3] |
| --- | --- |
| **Reference** | Notarnicola et al. (2010) |
| **Type** | Case-control study |
| **Patients** | 118 total  ESWT group: 58 (5♀, 53♂); mean age 33.2 years  Surgery group: 60 (8♀, 52♂); mean age 33 years |
| **Definition of nonunion** | “Cases that failed to show radiographic consolidation after at least 6 months of conservative treatment, persisting for a further three months.” |
| **Treatment site** | 118 scaphoid bones |
| **fESWT protocol / comparator** | ESWT group: three sessions; 72 h interval; 4,000 fESWs @ ø0.09 ± 0.02 mJ/mm^2^ (range, 0.05-0.12)  Comparator group: Matti-Russe surgery technique using bone grafts from ipsilateral olecranum |
| **fESWT device** | Minilith (electromagnetic) (Storz Medical) |
| **Anesthesia** | Not specified |
| **Interval** | ESWT group ø14.8 ± 6.9 months (range, 9-36)  Surgery group ø15.8 ± 7.5 months (range, 9-40) |
| **Follow-up** | 2, 6 and 12 months |
| **Outcome** | Equal to comparator  ESWT group: union in 44/58 (75.9%) of patients after 2 months, and in 46/58 (79.3%) of patients after 6 and 12 months  Surgery group: union in 46/60 (76.7%) patients after 2 months, and in 47/60 (78.3%) of patients after 6 and 12 months |

**3) Case series (no control group)**

| **Reference no.** | [4] |
| --- | --- |
| **Reference** | Valchanou & Michailov (1991) |
| **Type** | Case series |
| **Patients** | 79 (8♀, 71♂); mean age 28 years (range, 9-76) |
| **Definition of nonunion** | Not specified |
| **Treatment sites** | 79 including radius (21), scaphoid (17), ulna (11), tibia (10), phalanx (7), femur (6), humerus (5), patella (1) and fibula (1) |
| **fESWT protocol** | Single session; 1,000-4,000 fESWs @ bone focus 1,000-1,700 bars (not further specified) |
| **fESWT device** | Not specified (electrohydraulic) |
| **Anesthesia** | Regional (upper limb), epidural (lower limb) |
| **Interval** | On average 20.2 months |
| **Follow-up** | Not specified (presumably from 3 months to 2 years after fESWT)  Cast for averaged 81 days (range, 20-120) |
| **Outcome** | Union in 70/82 (85,4%) of patients  Unsuccessful: femur (1), scaphoid (4), tibia (3), radius (2), ulna (1), patella (1) |

| **Reference no.** | [5] |
| --- | --- |
| **Reference** | Schleberger & Senge (1992) |
| **Type** | Case series |
| **Patients** | 4 (2♀, 2♂); mean age 17.25 years (range, 3-34) |
| **Definition of nonunion** | "Non-union for at least 5 months after initial treatment of the fracture." |
| **Treatment sites** | 4 including tibia (2), diaphysis in humerus midshaft (1) and 5^th^ metatarsus |
| **fESWT protocol** | Single session; 2,000 fESWs @ 18 kV |
| **fESWT device** | MFL 5000 and HM3 (electrohydraulic) (Dornier MedTech) |
| **Anesthesia** | General (3) and plexus (1) |
| **Interval** | ≥5 months |
| **Follow-up** | Most probably 4 months |
| **Outcome** | Union in 3/4 (75%) of patients; unsuccessful in case of a tibia |

| **Reference no.** | [6] |
| --- | --- |
| **Reference** | Heinrichs et al. (1993) |
| **Type** | Case series |
| **Patients** | 53 (not further specified) |
| **Definition of nonunion** | “Fehlende Knochenheilung nach 6 Monaten oder eine deutlich hinter dem normalen Heilungsverlauf zurückliegende Knochenheilung.” [“A lack of bone healing after 6 months or a bone healing significantly behind the normal healing process.”] |
| **Treatment sites** | Various nonunion sites including radius, carpal arthrodesis, scaphoid, femur, tibia, talus and metatarsus |
| **fESWT protocol** | Presumably single session, 1,500-10,000 fESWs, energy flux density not specified |
| **fESWT device** | Lithostar and Osteostar (electromagnetic) (Siemens, Erlangen, Germany) |
| **Anesthesia** | Regional mostly, one general |
| **Interval** | Not specified |
| **Follow-up** | Not specified |
| **Outcome** | Union in 67% of patients |

| **Reference no.** | [7] |
| --- | --- |
| **Reference** | Diesch and Haupt (1997) |
| **Type** | Case series |
| **Patients** | 172 (57♀, 115♂); mean age 41 ± 15.4 years (range, 7-79) |
| **Definition of nonunion** | “Kommt eine Fraktur in einem Zeitraum von 6 Monaten nicht zur Durchbauung, so wird dies als *Pseudarthrose* definiert.” [“Healing of a fracture that does not appear within 6 months is called pseudarthrosis.”] |
| **Treatment sites** | 172 including lower leg (65), foot (38), femur (36), forearm (18), humerus (14) and hand (1) |
| **fESWT protocol** | Single session; 2,000-3,000 fESWs @ 0.3-0.35 mJ/mm^2^ |
| **fESWT device** | MFL 5000 (electrohydraulic) (Phillips, Eindhoven, The Netherlands), Ossatron (electrohydraulic) (HMT) and Compact S (electromagnetic) (Dornier MedTech) |
| **Anesthesia** | General |
| **Interval** | Not specified |
| **Follow-up** | 3 months |
| **Outcome** | Union in 66% of patients after 3-6 mo |

| **Reference no.** | [8] |
| --- | --- |
| **Reference** | Haupt (1997a) |
| **Type** | Case series |
| **Patients** | >100 |
| **Definition of nonunion** | Not specified |
| **Treatment sites** | Not specified |
| **fESWT protocol** | Not specified |
| **fESWT device** | Not specified (most probably electrohydraulic) |
| **Anesthesia** | Not specified |
| **Interval** | Not specified |
| **Follow-up** | Not specified |
| **Outcome** | Union in 65% of patients, and in 76% after exclusion of patients with scaphoid or atrophic pseudoarthrosis |

| **Reference no.** | [9] |
| --- | --- |
| **Reference** | Haupt (1997b) |
| **Type** | Case series |
| **Patients** | 87 |
| **Definition of nonunion** | "Healing that does not appear within 6 months is called pseudarthrosis." |
| **Treatment sites** | Not specified |
| **fESWT protocol** | Most probably single session; 1,000-3,000 fESWs @ 18-24 kV |
| **fESWT device** | MFL 5000 (electrohydraulic) (Phillips) and Ossatron (HMT) (electrohydraulic) |
| **Anesthesia** | Plexus for upper limb and peridural, spinal or general for lower limb |
| **Interval** | Not specified |
| **Follow-up** | Not specified |
| **Outcome** | Union in 67% of patients, and in 76% after exclusion of patients with scaphoid or atrophic pseudoarthrosis with insufficient stabilization |

| **Reference no.** | [10] |
| --- | --- |
| **Reference** | Vogel et al. (1997a) |
| **Type** | Case series |
| **Patients** | 52 (20♀, 32♂); mean age 37 years (range, 12-81) |
| **Definition of nonunion** | " Bone healing disorders which had existed for at least 6 months since the initial trauma [Knochenheilungsstörungen, die seit dem initialen Trauma mindestens 6 Monate bestanden]." |
| **Treatment sites** | 52 including lower extremity in 85% of patients; 8 patients with previously infected pseudoarthrosis |
| **fESWT protocol** | Not specified |
| **fESWT device** | Not specified |
| **Anesthesia** | Not specified |
| **Interval** | Not specified |
| **Follow-up** | Not specified |
| **Outcome** | Union in 27/52 (52%) of patients after averaged 3.3 months (range, 2-9); unsuccessful in cases of atrophic pseudarthrosis, fibrous dysplasia and ostegenesis imperfecta, and in many cases of scaphoid pseudarthrosis |

| **Reference no.** | [11] |
| --- | --- |
| **Reference** | Vogel et al. (1997b) |
| **Type** | Case series |
| **Patients** | 48 (23♀, 25♂); mean age 38 years (range, 12-81) |
| **Definition of nonunion** | "Only non-unions with a history of more than 6 months were included." |
| **Treatment sites** | 48 including tibia (19), femur (17), metatarsus (10), knee (1) and ankle (1) |
| **fESWT protocol** | Single session, 3,000 fESWs @ 0.6 mJ/mm^2^ |
| **fESWT device** | Osteostar (electromagnetic) (Siemens) |
| **Anesthesia** | Regional except for 1 child |
| **Interval** | On average 12 months (range, 6-48) |
| **Follow-up** | 3, 6 and 12 months |
| **Outcome** | Union in 29/48 (60.4%) of patients after averaged 3.4 months (range, 2-9) |

| **Reference no.** | [12] |
| --- | --- |
| **Reference** | Beutler et al. (1999) |
| **Type** | Case series |
| **Patients** | 25; mean age 35 years (range, 19-72) |
| **Definition of nonunion** | "A pseudoarthrosis occurs when there are no radiographic signs of healing 6 months after fracture, and pain or pathological mobility occurs during clinical bending or rotational stress [Eine Pseudarthrose liegt vor, wenn 6 Monate nach Fraktur keine röntgenologischen Anzeichen einer Ausheilung vorliegen und Schmerzen oder eine pathologische Beweglichkeit bei klinischer Biege- oder Rotationsbelastung ("Streßtest") auftreten]." |
| **Treatment sites** | 27 including tibia (11), femur (9), ulna (5) and humerus (2) |
| **fESWT protocol** | Two sessions, two week interval; 2,000 fESWs @ 18 kV per session |
| **fESWT device** | MFL 5000 (electrohydraulic) (Dornier MedTech) |
| **Anesthesia** | None except for one case |
| **Interval** | On average 9 months (range, 6-16) |
| **Follow-up** | 6 months |
| **Outcome** | Union in 11/27 (41%) of patients; unsuccessful in cases of atrophic and/or metaphysal pseudoarthrosis |

| **Reference no.** | [13] |
| --- | --- |
| **Reference** | Rompe et al. (2001) |
| **Type** | Case series |
| **Patients** | 43 (20♀, 23♂); mean age 39.5 ± 8.5 years (range, 18-74) |
| **Definition of nonunion** | "A pseudarthrosis was diagnosed when a minimum of 9 months had elapsed since the last operation, and no radiologic bridging of the four cortices of bone was observed on the anteroposterior and lateral radiographs." |
| **Treatment sites** | 43 including femur (24) and tibia (19) |
| **fESWT protocol** | Single session; 3,000 fESWs @ 0.6 mJ/mm^2^ |
| **fESWT device** | Osteostar (electromagnetic) (Siemens) |
| **Anesthesia** | Regional |
| **Interval** | On average 11.4 ± 4.5 months (range, 9-36) |
| **Follow-up** | 9 months |
| **Outcome** | Union in 31/43 (72%) of patients after averaged 4 months (range, 2-7); among 12 unsuccessful cases were 6 heavy smokers |

| **Reference no.** | [14] |
| --- | --- |
| **Reference** | Schaden et al. (2001) |
| **Type** | Case series |
| **Patients** | 115 (41♀, 74♂); ♀: mean age 47.1 years (range, 15-85); ♂: mean age 40.6 years (range, 10-86) |
| **Definition of nonunion** | "The delay from the initial injury or the last operation was…more than 6 months… (nonunion)." |
| **Treatment sites** | 115 including long bone shaft (72) and other bones (43) |
| **fESWT protocol** | Single session; scaphoid bone 1,000-2,500 fESWs @ 0.25-0.35 mJ/mm^2^ (20-24 kV); tibia and femur 12,000 fESWs @ 0.4 mJ/mm^2^ (28 kV) |
| **fESWT device** | Not specified (electrohydraulic) |
| **Anesthesia** | General or regional (plexus and spinal) |
| **Interval** | On average >6 months (range, 3-300) |
| **Follow-up** | On average 18 months (range, 3-48) |
| **Outcome** | Union in 87/115 (75.7%) of patients; unsuccessful in cases where patients were unsuitable for ESWT (7.9%), received inadequate immobilization (1.7 %) or too early reoperation (1.7%), or experienced new trauma (0.8%); no reason for lack of success in 12.2% of patients |

| **Reference no.** | [15] |
| --- | --- |
| **Reference** | Wang et al. (2001) |
| **Type** | Case series |
| **Patients** | 72 (19♀, 53♂); mean age 39.4 years (range, 15-74) |
| **Definition of nonunion** | "Failure to show bony union 6 months after initial closed or open treatment." |
| **Treatment sites** | 72 long bone fractures incl. radius, ulna, humerus, femur and tibia |
| **fESWT protocol** | Single session; metatarsal fractures 1,000 fESWs @ 0.47 mJ/mm^2^ (20 kV); radius and ulna 2,000 fESWs @ 0.56 mJ/mm^2^ (24 kV); humerus 3,000 fESWs @ 0.56 mJ/mm^2^ (24 kV); femur and tibia 6,000 fESWs @ 0.62 mJ/mm^2^ (28 kV) |
| **fESWT device** | Ossatron (electrohydraulic) (HMT) |
| **Anesthesia** | General or spinal |
| **Interval** | Not specified |
| **Follow-up** | 3 months (70 patients), 6 mo (61 patients) and 12 mo (55 patients) |
| **Outcome** | Union in 28/70 (40%) of patients after 3 months, in 37/61 (60.7%) after 6 months and in 44/55 (80%) after 12 months; least successful in atrophic unions (0/13 after 3 months, 3/11 after 6 months and 6/8 after 12 months) |

| **Reference no.** | [16] |
| --- | --- |
| **Reference** | Küfer et al. (2002) |
| **Type** | Case series |
| **Patients** | 4 (gender distribution and age not specified) |
| **Definition of nonunion** | "Lack of bony consolidation for at least 6 months." |
| **Treatment sites** | 4 (not further specified) |
| **fESWT protocol** | Three sessions; interval between sessions not specified; 2,500 fESWs @ >0.12 mJ/mm^2^ |
| **fESWT device** | C-150 (electromagnetic) (Dornier MedTech) |
| **Anesthesia** | Plexus |
| **Interval** | ≥6 months |
| **Follow-up** | Not specified |
| **Outcome** | Union in 3/4 (75%) of patients; unsuccessful in one case where bone scan before ESWT showed inactivity |

| **Reference no.** | [17] |
| --- | --- |
| **Reference** | Schatz et al. (2002) |
| **Type** | Case series |
| **Patients** | 31 |
| **Definition of nonunion** | Congenital tibial pseudarthrosis |
| **Treatment sites** | 31 delayed unions including congenital tibial pseudoarthrosis (4) |
| **fESWT protocol** | Single session, 3D navigated, 6,000 fESWs @ 1.5 mJ/mm^2^ |
| **fESWT device** | Lithotrack (electromagnetic) (Storz Medical) |
| **Anesthesia** | General |
| **Interval** | On average 10.5 months (7-16) |
| **Follow-up** | >12 months |
| **Outcome** | Union in 21/31 (67.7%) of patients after averaged 3.5 months |

| **Reference no.** | [18] |
| --- | --- |
| **Reference** | Biedermann et al. (2003) |
| **Type** | Case series |
| **Patients** | 73 (34♀, 39♂); mean age 42 years (range, 18-74) |
| **Definition of nonunion** | "Failed bone healing of more than 6 months." |
| **Treatment sites** | 70 including long bones (58), other bones (12); 3 patients were lost to follow-up |
| **fESWT protocol** | Single session (12/73 patients received two sessions); on average 2,900 fESWs @ averaged 23 kV (~0.7 mJ/mm^2^) |
| **fESWT device** | MFL 5000 (electrohydraulic) (Dornier MedTech) |
| **Anesthesia** | General or regional |
| **Interval** | ≥6 months |
| **Follow-up** | 3 months |
| **Outcome** | Union in 56% of patients |

| **Reference no.** | [19] |
| --- | --- |
| **Reference** | Chooi and Penafort (2004) |
| **Type** | Case series |
| **Patients** | 5 (1♀, 4♂); ♀: 40 years old; ♂: mean age 29.5 years (range, 19-48) |
| **Definition of nonunion** | "Failure of fracture union after a minimum of 6 months from initial injury, with no progression of radiographic healing for a minimum of 3 months before shock-wave treatment." |
| **Treatment sites** | 6 including femur mid shaft (5) and tibia mid shaft (1) |
| **fESWT protocol** | Single session; 4,000 fESWs @ 25 kV |
| **fESWT device** | Ossatron (electrohydraulic) (HMT) |
| **Anesthesia** | General or spinal |
| **Interval** | On average 26.6 months (range, 6-84) |
| **Follow-up** | On average 31 weeks (range, 17-38) |
| **Outcome** | Union in 2/5 (40%) of patients after 22 weeks |

| **Reference no.** | [20] |
| --- | --- |
| **Reference** | Schaden et al. (2004) |
| **Type** | Case series |
| **Patients** | 613 (196♀, 417♂); mean age 43.7 years (range, 10-90) |
| **Definition of nonunion** | "More than 6 months" |
| **Treatment sites** | 613 including tibia (181), scaphoid (85), femur (85), humerus (51), foot (51, incl. 2 patellae), ulna (42), hand (40), radius (36), fibula (19), upper ankle joint arthrodesis (11), pelvis (7) and femoral neck (5) |
| **fESWT protocol** | Single session; 2,000-4,000 fESWs @ 26 kV (~0.38 mJ/mm^2^) |
| **fESWT device** | Ossatron (electrohydraulic) (HMT) |
| **Anesthesia** | General, regional or local |
| **Interval** | On average 16.1 months |
| **Follow-up** | 6 months |
| **Outcome** | Union in 466/613 (76%) of patients |

| **Reference no.** | [21] |
| --- | --- |
| **Reference** | Bara and Snyder (2007) |
| **Type** | Case series |
| **Patients** | 81 (26♀, 55♂); age range 12-89 years |
| **Definition of nonunion** | Not specified ("The period between the fracture and ESWT has varied from  4 months to 17 years." |
| **Treatment sites** | 81 including tibia (49), femur (13), radius & ulna (10), humerus (5) and other bones (4) |
| **fESWT protocol** | Unclear whether single or multiple sessions; larger bones 3,000 fESWs @ ~20 kV; forearm and smaller bones 1,500 fESWs @ ~20 kV |
| **fESWT device** | Econolith 2000 (electrohydraulic) (Medispec, Yehud, Israel) |
| **Anesthesia** | No anaesthesia |
| **Interval** | On average 8 months (range, 4 montsh – 17 years) |
| **Follow-up** | 6 months |
| **Outcome** | Union in 67/81 (83%) of patients |

| **Reference no.** | [22] |
| --- | --- |
| **Reference** | Taki (2007) |
| **Type** | Case series |
| **Patients** | 5 (1♀, 4♂) athletes; mean age 19 years (range, 17-22) |
| **Definition of nonunion** | Not provided ("The time interval between the initial symptoms and ESWT ranged  from 6 to 25 months (mean, 12 months)." |
| **Treatment sites** | 5 including tibia (2), inferior pubic ramus (1), medial malleolus (1) and base of 5^th^ metatarsus (1) |
| **fESWT protocol** | Single session, 2,000-4,000 fESWs @ 22-28 kV (0.29-0.4 mJ/mm^2^); |
| **fESWT device** | Ossatron (electrohydraulic) (HMT) |
| **Anesthesia** | Spinal |
| **Interval** | On average 12 months (range, 6-25) |
| **Follow-up** | Not specified |
| **Outcome** | Union in 5/5 (100%) of patients after averaged 2.9 months |

| **Reference no.** | [23] |
| --- | --- |
| **Reference** | Endres et al. (2008) |
| **Type** | Case series |
| **Patients** | 1 (♂); 23 years old |
| **Definition of nonunion** | "Pseudoarthrosis… (12 weeks postoperative)." |
| **Treatment site** | Complicated tibia-fibular shaft fracture |
| **fESWT protocol** | Four sessions within six weeks; number of fESWs not specified; 0.4 mJ/mm^2^ |
| **fESWT device** | Duolith SD1 (electromagnetic) (Storz Medical) |
| **Anesthesia** | Not specified |
| **Interval** | Approximately 9 months |
| **Follow-up** | Not specified |
| **Outcome** | Union after ≤8 weeks |

| **Reference no.** | [24] |
| --- | --- |
| **Reference** | Cacchio et al. (2009b) |
| **Type** | Case series |
| **Patients** | 34 (8♀, 26♂); mean age 42.5 ± 5.9 years |
| **Definition of nonunion** | Not specified. |
| **Treatment sites** | 34 including tibia (22) and femur (12) |
| **fESWT protocol** | Four sessions @ one-week intervals; 4,000 fESWs @ 0.40 mJ/mm^2^ |
| **fESWT device** | Modulith SLK (electromagnetic) (Storz Medical) |
| **Anesthesia** | Not specified |
| **Interval** | Not specified |
| **Follow-up** | 6 months |
| **Outcome** | Union in 26/34 (76.5%) of patients |

| **Reference no.** | [25] |
| --- | --- |
| **Reference** | Moretti et al. (2009) |
| **Type** | Case series |
| **Patients** | 204 (gender distribution and age not specified) |
| **Definition of nonunion** | Not specified. |
| **Treatment sites** | Not specified |
| **fESWT protocol** | Single session; 4,000 fESWs @ 0.22-1.1 mJ/mm^2^ |
| **fESWT device** | Minilith (electromagnetic) (Storz Medical) |
| **Anesthesia** | Not specified |
| **Interval** | Not specified |
| **Follow-up** | 3 months |
| **Outcome** | Union in 174/204 (85%) of patients |

| **Reference no.** | [26] |
| --- | --- |
| **Reference** | Wang et al. (2009) |
| **Type** | Case series |
| **Patients** | 42 (20♀, 22♂); mean age 34.8 ± 13.6 years (range, 18-68) |
| **Definition of nonunion** | "Fracture failed to heal in 6 months from the initial treatment." |
| **Treatment sites** | 42 including femur (28) and tibia (14) |
| **fESWT protocol** | Single session; 6,000 fESWs @ 28 kV (0.62 mJ/mm^2^) |
| **fESWT device** | Ossatron (electrohydraulic) (Sanuwave, Alpharetta, GA, USA) |
| **Anesthesia** | General |
| **Interval** | On average 15 ± 10.8 months (range, 6-48) |
| **Follow-up** | 6 months |
| **Outcome** | Union in 33/42 (78.6%) of patients |

| **Reference no.** | [27] |
| --- | --- |
| **Reference** | Xu et al. (2009) |
| **Type** | Case series |
| **Patients** | 69 (25♀, 44♂); mean age 38 ± 12.3 years (range, 22-72) |
| **Definition of nonunion** | "Diagnosis of nonunion was made by at least two post-op X-rays 3 months after the fracture or the previous operation." |
| **Treatment sites** | 69 including tibia (28), femur (22), humerus (13), radius (5) and ulna (1) |
| **fESWT protocol** | Single session; femur and tibia 6,000-10,000 fESWs @ 28 kV (0.62 mJ/mm^2^); humerus 4,000 fESWs @ 24 kV (0.56 mJ/mm^2^); radius and ulna 3,000 fESWs @ 24 kV (0.56 mJ/mm^2^) |
| **fESWT device** | Ossatron (electrohydraulic) (HMT) |
| **Anesthesia** | Spinal or local |
| **Interval** | On average 12.5 ± 10.3 months (range, 6-84) |
| **Follow-up** | 6 – 90 months |
| **Outcome** | Union in 65.2% of patients after 6 months and 75.8% after 90 months; unsuccessful in atrophic nonunions |

| **Reference no.** | [28] |
| --- | --- |
| **Reference** | Elster et al. (2010) |
| **Type** | Case series |
| **Patients** | 192 (172 until follow-up) (50♀, 122♂); mean age 44.6 ± 14.4 years (range, 16-90) |
| **Definition of nonunion** | "A fracture that: 1) has failed to demonstrate cortical continuity on three of four cortices despite operative or nonoperative intervention for 6 months or more; or 2) showed no radiographic changes for 3 consecutive months and was associated with inability to bear weight on the affected extremity, pain on palpation, or motion at the fracture site 6 months posttrauma." |
| **Treatment sites** | 192 tibiae |
| **fESWT protocol** | 153 patients with one session, 29 patients with two sessions, nine patients with three sessions, one patient with four sessions; on average 5,510 ± 3,610 fESWs (range, 2,000-12,000) @ 26-28 kV (0.38-0.40 mJ/mm^2^) |
| **fESWT device** | Ossatron (electrohydraulic) (HMT) |
| **Anesthesia** | General or regional |
| **Interval** | On average 16.8 ± 27.9 months |
| **Follow-up** | 6 months |
| **Outcome** | Union in 138/192 (71.9%) of patients |

| **Reference no.** | [29] |
| --- | --- |
| **Reference** | Alvarez et al. (2011) |
| **Type** | Case series |
| **Patients** | 32 (25♀, 7♂); on average 49.8 ± 15.8 years (range, 16-75) |
| **Definition of nonunion** | "Lack of progression or not healed at 24 weeks." |
| **Treatment sites** | 34 proximal metatarsal fractures |
| **fESWT protocol** | Single session; 2,000 fESWs @ 0.22-0.51 mJ/mm^2^ |
| **fESWT device** | Ossatron (electrohydraulic) (HMT) |
| **Anesthesia** | General or regional |
| **Interval** | On average 7 ± 4 months (range, 2.5 months - 16 years) |
| **Follow-up** | 3, 6 and 12 months |
| **Outcome** | Union in 29/31 (93.5%) of patients after 3 months (17 total unions = 54.8%, 12 partial unions = 38.7%); in 26/26 (100%) after 6 months (19 total = 73%, 7 partial = 27%); in 20/20 (100%) patients after 12 months (19 total = 95%, 1 partial = 5%) |

| **Reference no.** | [30] |
| --- | --- |
| **Reference** | Stojadinovic et al. (2011) |
| **Type** | Case series |
| **Patients** | 349 (114♀, 235♂); average age 48 ± 16 years (range, 15-91) |
| **Definition of nonunion** | “A fracture that has failed to show continuity of three of four cortices after surgical or nonsurgical treatment for six or more months from the time of the fracture-related injury, or has failed to demonstrate any radiographic change (improvement) for three consecutive months, and is associated with clinical findings consistent with a fracture nonunion (an inability to bear weight on the affected extremity, pain on palpation, or motion at the fracture site for three to six months or more following the incident traumatic event or the last surgical procedure.” |
| **Treatment sites** | 349 including tibial shaft (106), femoral shaft (70), humerus (38), hand (34), ulna (29), foot (28), radius (21), patella (11), pelvis (7) and fibular shaft (5) |
| **fESWT protocol** | Single session in 273/349 (78.2%) of patients; >1 session in 56/349 (16.1%) of patients XXX |
| **fESWT device** | Orthowave 280 (electrohydraulic) (TRT, Woodstock, GA, USA) |
| **Anesthesia** | General or regional |
| **Interval** | ≤6 months in case of 120/349 (34.4%) patients; 6-11.3 months in case of 116/349 (33.2%) patients; >11.3 months in case of 113/349 (32.4%) patients |
| **Follow-up** | 6 months |
| **Outcome** | Union in 94/106 (88.7%) of tibial shafts, 51/70 (72.9%) of femoral shafts, 28/28 (100%) of feet, 25/38 (65.8%) of humeri, 24/34 (70.6%) of hands, 22/29 (75.9%) of ulnae, 18/21 (85.7%) of radii, 9/11 (81.8%) of patellae, 6/7 (85.7%) of pelves and 5/5 (100%) of fibular shafts |

| **Reference no.** | [31] |
| --- | --- |
| **Reference** | Vulpiani et al. (2012) |
| **Type** | Case series |
| **Patients** | 143 (51♀, 92♂); mean age 41.4 years (range, 14-81) |
| **Definition of nonunion** | "A fracture that did not demonstrate cortical continuity on radiographs despite operative or nonoperative intervention for 6 months." |
| **Treatment sites** | 143 including tibia (39), humerus (25), femur (24), scaphoid (17), ulna (13), radius (7), fibula (7), collarbone (6) and metatarsus (5) |
| **fESWT protocol** | 96 patients with one treatment cycle, 39 patients with two treatment cycles and eight patients with three treatment cycles @ 3 months intervals; each cycle with 3-5 sessions @ 48-72 h intervals and 2,500-3,000 fESWs @ 0.25-0.84 mJ/mm^2^ depending on bone size |
| **fESWT device** | Device from Storz Medical (electromagnetic) (not further specified) |
| **Anesthesia** | Scaphoids with local anaesthesia, other bones without anaesthesia |
| **Interval** | On average 14.1 months (range, 6-84) |
| **Follow-up** | 12 months |
| **Outcome** | Union in 80/143 (55.9%) of patients after averaged 7.6 months (range, 2-24); unsuccessful mainly in atrophic nonunions |

| **Reference no.** | [32] |
| --- | --- |
| **Reference** | Czarnowska-Cubała et al. (2013) |
| **Type** | Case series |
| **Patients** | 31 (11♀, 20♂); ♀: mean age 51 ± 3 years (range, 31-68); ♂: mean age 45 ± 4 years (range, 21-72) |
| **Definition of nonunion** | Not specified. |
| **Treatment sites** | 31 including humerus (8), tibia (8), radius (5), ulna (5), femur (4) and metatarsus (1) |
| **fESWT protocol** | Single session; 3,000 fESWs @ 20-21 kV |
| **fESWT device** | Econolith 2000 (electrohydraulic) (Medispec) |
| **Anesthesia** | Not specified |
| **Interval** | On average 22.6 months |
| **Follow-up** | 6 months |
| **Outcome** | Complete union in 12/31 (38.7%) of patients |

| **Reference no.** | [33] |
| --- | --- |
| **Reference** | Alkhawashki (2015) |
| **Type** | Case series |
| **Patients** | 44 (9♀, 35♂); mean age 34 years (range, 14-70) |
| **Definition of nonunion** | "Type A1 and 2 nonunion, according to Weber and Cech [34] and patients who had nonunion after surgical procedures; i.e., osteotomies, as well as those previously treated, either non-operatively or by open reduction internal fixation (ORIF)." |
| **Treatment sites** | 49 including femur shaft (22), tibia shaft (8), femur neck (5), humerus (4), scaphoid (4), high tibial osteotomy (2), collarbone (1), base of 5^th^ metatarsus (1), sub-trochanteric osteotomy (1) and inter-trochanteric osteotomy (1) |
| **fESWT protocol** | 38 bones with one session, nine bones with two sessions, two bones with three sessions; 12-week intervals between sessions; larger bones with 3,000-4,000 fESWs @ 26 kV; smaller bones with 2,000-3,000 fESWs @ 26 kV |
| **fESWT device** | Ossatron (electrohydraulic) HMT |
| **Anesthesia** | Not specified |
| **Interval** | On average 11.9 months (range, 6-60) |
| **Follow-up** | On average 18 months (range, 3-19) |
| **Outcome** | Union in 37/49 (75.5%) of patients after averaged 10.2 months (range, 3-19); unsuccessful in cases involving low grade infection (4 femoral shafts), >5mm gap size (4 femoral shafts), comprised vascularity (2 scaphoids, 1 femur neck) and instability (1 humerus neck) |

| **Reference no.** | [34] |
| --- | --- |
| **Reference** | Kuo et al. (2015) |
| **Type** | Case series |
| **Patients** | 22 (9♀, 13♂); mean age 30 years (range, 18-45) |
| **Definition of nonunion** | "A fracture that failed to achieve cortical continuity on three of four cortices after initial nailing for 6 months or more; or 2) a fracture that showed no radiographic progress towards union for 3 consecutive months and was associated with  inability to bear weight on the affected extremity, pain on palpation, or motion at the fracture site 6 months after the initial trauma." |
| **Treatment sites** | 22 atrophic nonunions of isthmic femoral shaft fractures |
| **fESWT protocol** | Single session; 6,000 fESWs @ 28 kV (0.58 mJ/mm^2^) |
| **fESWT device** | Ossatron (electrohydraulic) (Sanuwave) |
| **Anesthesia** | Not specified |
| **Interval** | On average 10.5 months (range, 6-16) |
| **Follow-up** | 12 months |
| **Outcome** | Union in 14/22 (63.6%) of patients |

| **Reference no.** | [35] |
| --- | --- |
| **Reference** | Haffner et al. (2016) |
| **Type** | Case series |
| **Patients** | 52 (12♀, 40♂); mean age 49 years (range, 15-85) |
| **Definition of nonunion** | "Non-union is established when a *[1]* fracture fails to demonstrate cortical continuity despite operative and non-operative interventions for a period of 9 or more months; and, *[2]* fracture fails to demonstrate any sign of healing on  radiographic assessment of the fracture in the following 3 months."  (Note that *[1]* refers to [38] and *[2]* refers to Elster et al., 2010 [28] outlined above. However, the definition of non-union provided in [28] is different from the definition of non-union provided here.) |
| **Treatment sites** | Tibia |
| **fESWT protocol** | Single session; 3000-4000 fESWs @ 0.4 mJ/mm^2^ |
| **fESWT device** | OrthoGold280 (electrohydraulic) (MTS) |
| **Anesthesia** | General or regional anaesthesia (spinal block) |
| **Interval** | On average 15.6 months |
| **Follow-up** | 6 months |
| **Outcome** | Union in 46/52 (88.5%) of patients |

**3) Case series performed with extracorporeal shock waves generated by means of explosions**

| **Reference no.** | [36] |
| --- | --- |
| **Reference** | Ikeda et al. (1999) |
| **Type** | Case series |
| **Patients** | 6 (2♀, 4♂); mean age 38.6 years (range, 21-76) |
| **Definition of nonunion** | Not specified. |
| **Treatment site** | 6 including tibia (3), humerus (1), radius (1) and femur (1) |
| **fESWT protocol** | Presumably single session, on average 282 fESWs (range, 100-800) @ 10-40 mJ/mm^2^ |
| **Anesthesia** | General or nerve block |
| **Interval** | On average approximately 14 months (range, 5-36) |
| **Follow-up** | Not specified |
| **Outcome** | Union in 4/6 (66.6%) of patients after 4.3 months |

| **Reference no.** | [37] |
| --- | --- |
| **Reference** | Ikeda (2009) |
| **Type** | Case series |
| **Patients** | 8 (gender distribution and age not specified) |
| **Definition of nonunion** | Not specified. |
| **Treatment site** | 8 including tibia (4), humerus (2), radius (1) and femur (1) |
| **fESWT protocol** | Presumably single session; 300-400 fESWs @ peak pressure of 300 MPa (1.0 J) |
| **Anesthesia** | Not specified (epidural in at least one case) |
| **Interval** | Not specified |
| **Follow-up** | Not specified |
| **Outcome** | Union in 5/8 (62.5%) of patients after averaged 4.3 months |

**References**

1. Cacchio A, Giordano L, Colafarina O, Rompe JD, Tavernese E, Ioppolo F, et al. Extracorporeal shock-wave therapy compared with surgery for hypertrophic long-bone nonunions. J Bone Joint Surg Am. 2009a;91:2589-97.
2. Furia JP, Juliano PJ, Wade AM, Schaden W, Mittermayr R. Shock wave therapy compared with intramedullary screw fixation for nonunion of proximal fifth metatarsal metaphyseal-diaphyseal fractures. J Bone Joint Surg Am. 2010;92:846-54.
3. Notarnicola A, Moretti L, Tafuri S, Gigliotti S, Russo S, Musci L, et al. Extracorporeal shockwaves versus surgery in the treatment of pseudoarthrosis of the carpal scaphoid. Ultrasound Med Biol. 2010;36:1306-13.
4. Valchanou VD, Michailov P. High energy shock waves in the treatment of delayed and nonunion of fractures. Int Orthop. 1991;15:181-4.
5. Schleberger R, Senge T. Non-invasive treatment of long-bone pseudarthrosis by shock waves (ESWL(R)). Arch Orthop Trauma Surg. 1992;111:224-7.
6. Heinrichs W, Witzsch U, Bürger RA. Extrakorporale Stosswellentherapie (ESWT) von Pseudoarthrosen. Eine neue Indikation fur Regionalanasthesien. [Extracorporeal shock-wave therapy (ESWT) for pseudoarthrosis. A new indication for regional anesthesia]. Anaesthesist 1993;42:361-4.
7. Diesch R, Haupt G. Anwendung der hochenergetischen extrakorporalen Stosswellentherapie bei Pseudarthrosen [Use of extracorporeal shock waves in the treatment of pseudoarthrosis]. Orthop Praxis 1997:7:470-1.
8. Haupt G. Stosswellen in der Orthopädie [Shock waves in orthopedics]. Urologe 1997a;36:233-8.
9. Haupt G. Use of extracorporeal shock waves in the treatment of pseudarthrosis, tendinopathy and other orthopedic diseases. J Urol. 1997b;158:4-11.
10. Vogel J, Rompe JD, Hopf C, Heine J, Burger R. Die hochenergetische extrakorporale Stosswellentherapie (ESWT) in der Behandlung von Pseudarthrosen. [High-energy extracorporeal shock-wave therapy (ESWT) in the treatment of pseudarthrosis]. Z Orthop Ihre Grenzgeb. 1997a;135:145-9.
11. Vogel J, Hopf C, Eysel P, Rompe JD. Application of extracorporeal shock-waves in the treatment of pseudarthrosis of the lower extremity. Preliminary results. Arch Orthop Trauma Surg. 1997b;116:480-3.
12. Beutler S, Regel G, Pape HC, Machtens S, Weinberg AM, Kremeike I, et al. Extracorporeal shock wave therapy for delayed union of long bone fractures - Preliminary results of a prospective cohort study. Unfallchirurg 1999;102:839-47.
13. Rompe JD, Rosendahl T, Schollner C, Theis C. High-energy extracorporeal shock wave treatment of nonunions. Clin Orthop Rel Res. 2001;387:102-11.
14. Schaden W, Fischer A, Sailler A. Extracorporeal shock wave therapy of nonunion or delayed osseous union. Clin Orthop Relat Res. 2001;387:90-4.
15. Wang CJ, Chen HS, Chen CE, Yang KD. Treatment of nonunions of long bone fractures with shock waves. Clin Orthop Rel Res. 2001;(387):95-101.
16. Küfer R, Volkmer B, Nesslauer T, Schneider P, Hautmann RE, Gschwend JE. An innovative mobile lithotripter for extracorporeal shock wave lithotripsy and therapy. Urol Int. 2002;69:17-20.
17. Schatz KD, Nehrer S, Dorotka R, Kotz R. 3D-navigierte hochenergetische Stoßwellentherapie und Achskorrektur nach fehlgeschlagener Distraktionsbehandlung bei kongenitaler Tibiapseudarthrose [3D-navigated high energy shockwave therapy and axis correction after failed distraction treatment of congenital tibial pseudarthrosis]. Orthopade 2002;31:663-6.
18. Biedermann R, Martin A, Handle G, Auckenthaler T, Bach C, Krismer M. Extracorporeal shock waves in the treatment of nonunions. J Trauma 2003;54:936-42.
19. Chooi YS, Penafort R. Extra-corporeal shock-wave therapy in the treatment of non-unions. Med J Malaysia 2004;59:674-77.
20. Schaden W, Fischer A, Sailler A. Extrakorporale Stoßwellentherapie (ESWT) aus der Sicht der Traumatologie [Extracorporeal shock-wave therapy from viewpoint of traumatology]. J Mineralstoffwechsel 2004;11:40-5.
21. Bara T, Synder M. Nine-year experience with the use of shock waves for treatment of bone union disturbances. Ortop Traumatol Rehabil. 2007;9:254-8.
22. Taki M, Iwata O, Shiono M, Kimura M, Takagishi K. Extracorporeal shock wave therapy for resistant stress fracture in athletes: a report of 5 cases. Am J Sports Med. 2007;35:1188-92.
23. Endres S, Weiskirch M, Hinz C, Hütter F, Wilke A. Extracorporeal shock-wave therapy in the treatment of pseudoarthrosis: a case series. Cases J. 2008;1:276.
24. Cacchio A, De Blasis E, Rosa F, De Blasis D, de Paulis F, Santilli V, et al. Response of bone turnover biochemical markers to extracorporeal shock wave therapy in the management of long-bone nonunions. Clin Chem. 2009b;55:195-6.
25. Moretti B, Notarnicola A, Moretti L, Patella S, Tato I, Patella V. Bone healing induced by ESWT. Clin Cases Miner Bone Metab. 2009;6:155-8.
26. Wang CJ, Yang KD, Ko JY, Huang CC, Huang HY, Wang FS. The effects of shockwave on bone healing and systemic concentrations of nitric oxide (NO), TGF-(beta)1, VEGF and BMP-2 in long bone non-unions. Nitric Oxide 2009;20:298-303.
27. Xu ZH, Jiang Q, Chen DY, Xiong J, Shi DQ, Yuan T, et al. Extracorporeal shock wave treatment in nonunions of long bone fractures. Int Orthop. 2009;33:789-93.
28. Elster EA, Stojadinovic A, Forsberg J, Shawen S, Andersen RC, Schaden W. Extracorporeal shock wave therapy for nonunion of the tibia. J Orthop Trauma 2010;24:133-41.
29. Alvarez RG, Cincere B, Channappa C, Langerman R, Schulte R, Jaakkola J, et al. Extracorporeal shock wave treatment of non-or delayed union of proximal metatarsal fractures. Foot Ankle Int. 2011;32:746-54.
30. Stojadinovic A, Kyle Potter B, Eberhardt J, Shawen SB, Andersen RC, Forsberg JA, et al. Development of a prognostic naive bayesian classifier for successful treatment of nonunions. J Bone Joint Surg Am. 2011;93:187-94.
31. Vulpiani MC, Vetrano M, Conforti F, Minutolo L, Trischitta D, Furia JP, et al. Effects of extracorporeal shock wave therapy on fracture nonunions. Am J Orthop. 2012;41:E122-7.
32. Czarnowska-Cubała M, Gwoździewicz K, Studniarek M, Lasek J. Predictive role of scintigraphy (BS) in bone union induction using extracorporeal shock wave treatment (ESWT). J Orthop. 2013;10:70-3.
33. Alkhawashki HM. Shock wave therapy of fracture nonunion. Injury 2015;46:2248-52.
34. Kuo SJ, Su IC, Wang CJ, Ko JY. Extracorporeal shockwave therapy (ESWT) in the treatment of atrophic non-unions of femoral shaft fractures. Int J Surg. 2015;24(Pt B):131-4.
35. Haffner N, Antonic V, Smolen D, Slezak P, Schaden W, Mittermayr R, Stojadinovic A. Extracorporeal shockwave therapy (ESWT) ameliorates healing of tibial fracture non-union unresponsive to conventional therapy. Injury. 2016;47:1506-13.
36. Ikeda K, Tomita K, Takayama K. Application of extracorporeal shock wave on bone: preliminary report. J Trauma 1999;47:946-50.
37. Ikeda K. Bone fracture and the healing mechanisms. Application of the extracorporeal shock wave on treatment of fracture Clin Calcium 2009;19:718-25.
